# Effects of the anticonvulsant Galonal during long-term alcohol exposure

**DOI:** 10.1192/j.eurpsy.2025.970

**Published:** 2025-08-26

**Authors:** E. Markova, I. Savkin, M. Knyazheva

**Affiliations:** 1 Neuroimmunology Laboratory, State Research Institute of Fundamental and Clinical Immunology, Novosibirsk, Russian Federation

## Abstract

**Introduction:**

Galonal increases gamma-aminobutyric acid (GABA) neurotransmission in the brain by having a modulatory effect on neuronal GABAA-receptors (GABAA-R). The presence of functional GABAA-R on the surface of immune cells, in particular T-lymphocytes, which mediate modulation of the cell’s functional activity has also been described. Chronic alcohol use is associated with significant T-lymphocytes dysregulation within the adaptive immune system. It suggests that synthetic GABAA-R ligand Galonal, similar to its effects on neuronal cells, may cause modulation of the functional activity of the lymphocytes, thereby influencing the intensity of the immune response.

**Objectives:**

Considering the fact that GABAA-R proved to be the molecular targets of ethanol on the immune and nervous cells, we investigated behavior and immunomodulatory effects of the artificial GABA receptor ligand Galonal during long-term alcohol exposure to find new perspective pharmacological substances in the treatment of alcoholism.

**Methods:**

Galonal (100 mg/kg) was administered in mice with 6-month 10% ethanol exposure (suspension of 1% starch mucus intragastrically) for 10 days, after which animal’s alcohol consumption, behavior and immune parameters were estimated.

**Results:**

After the course of Galonal administration a decrease in alcohol motivation and stimulation of exploratory behavior have been established in long-term alcoholized mice. An increase in the humoral immune response was also recorded, assessed by the absolute and relative numbers of AFC, to a level characteristic of healthy animals of the corresponding age. Significant stimulation of the cellular immune response, estimated by the DTH reaction and lymphocytes proliferative activity was also registered.

**Conclusions:**

Galonal demonstrated positive neuroimmunomodulation effect during long-term alcohol exposure, therefore, its promising for clinical use in the treatment of alcoholism.

**Disclosure of Interest:**

None Declared

